# Elevated amygdala responses to emotional faces in youths with chronic irritability or bipolar disorder^☆^

**DOI:** 10.1016/j.nicl.2013.04.007

**Published:** 2013-04-21

**Authors:** Laura A. Thomas, Pilyoung Kim, Brian L. Bones, Kendra E. Hinton, Hannah S. Milch, Richard C. Reynolds, Nancy E. Adleman, Abigail A. Marsh, R.J.R. Blair, Daniel S. Pine, Ellen Leibenluft

**Affiliations:** a Emotion and Development Branch, National Institute of Mental Health, National Institutes of Health, Department of Health and Human Services, Bethesda, MD, USA; b National Institute on Drug Abuse, National Institutes of Health, Department of Health and Human Services, Bethesda, MD, USA; c Department of Psychology, University of Denver, Denver, CO, USA; d Boston University School of Medicine, Boston, MA, USA; e Scientific and Statistical Computing Core, National Institute of Mental Health, National Institutes of Health, Department of Health and Human Services, Bethesda, MD, USA; f Department of Psychology, Georgetown University, Washington D.C., USA

**Keywords:** Severe mood dysregulation, Bipolar disorder, Amygdala, Face, Emotion

## Abstract

A major controversy in child psychiatry is whether bipolar disorder (BD) presents in children as severe, non-episodic irritability (operationalized here as severe mood dysregulation, SMD), rather than with manic episodes as in adults. Both classic, episodic BD and SMD are severe mood disorders characterized by deficits in processing emotional stimuli. Neuroimaging techniques can be used to test whether the pathophysiology mediating these deficits are similar across the two phenotypes. Amygdala dysfunction during face emotion processing is well-documented in BD, but little is known about amygdala dysfunction in chronically irritable youth. We compared neural activation in SMD (n = 19), BD (n = 19), and healthy volunteer (HV; n = 15) youths during an implicit face-emotion processing task with angry, fearful and neutral expressions. In the right amygdala, both SMD and BD exhibited greater activity across all expressions than HV. However, SMD and BD differed from each other and HV in posterior cingulate cortex, posterior insula, and inferior parietal lobe. In these regions, only SMD showed deactivation in response to fearful expressions, whereas only BD showed deactivation in response to angry expressions. Thus, during implicit face emotion processing, youth with BD and those with SMD exhibit similar amygdala dysfunction but different abnormalities in regions involved in information monitoring and integration.

## Highlights

• Youths with severe mood dysregulation (SMD), bipolar disorder (BD), controls.
• Implicit face-emotion processing fMRI task with angry, fearful, neutral emotions.
• In R amygdala, SMD and BD had greater activity across all expressions vs controls.
• In whole brain analysis SMD had decreased, BD increased activity vs. other groups.

## 1 Introduction

The diagnosis of bipolar disorder (BD) is being assigned to youth with increasing frequency (Moreno et al., 2007). This upsurge may reflect the controversial view that mania manifests in youth as severe, non-episodic irritability (Leibenluft et al., 2003) rather than with distinct manic episodes, as in adults. To facilitate research on this question, we operationalized severe, chronic irritability in youth as “*severe mood dysregulation* (*SMD*)” to test whether it is distinct from episodic BD (Leibenluft et al., 2003). This question is important because severe irritability occurs much more commonly in youth than classic, episodic mania (Brotman et al., 2006), and because the diagnostic formulation has treatment implications (Leibenluft, 2011). Research thus far indicates that BD and SMD exhibit different family history and long-term clinical outcomes (i.e., children with severe irritability are at increased risk for developing unipolar depressive or anxiety disorders, rather than mania, in adulthood (Brotman et al., 2006; Stringaris et al., 2009)). However, it is important to complement research based on clinical measures with pathophysiological comparisons of the two clinical syndromes to ascertain the extent to which they share common neural deficits and hence are likely to respond to similar therapeutic interventions.

Here we compared neural circuitry perturbations in SMD and BD youths during face emotion processing. We focused on face emotion processing because both pediatric BD and SMD youth manifest behavioral deficits in this domain; both patient groups misidentify facial emotions, including happy, sad, fearful and angry expressions (Guyer et al., 2007; McClure et al., 2005; Rich et al., 2008; Schenkel et al., 2007). In child and adult BD, amygdala hyperactivity appears to occur in response to fearful, angry, happy, and neutral faces (Chen et al., 2011; Kalmar et al., 2009; Kim et al., 2012; Pavuluri et al., 2007). However, only two prior studies focus on neural deficits during face emotion information processing in SMD. The first found differences in amygdala activation between SMD vs. both BD and healthy volunteers (HV) (Brotman et al., 2010). Specifically, SMD youths showed amygdala *deactivation* during explicit processing of their emotional response to neutral faces (i.e., rating how afraid they were of neutral faces) and *hyperactivity* during implicit processing of the neutral face (i.e., while rating nose-width). BD did not differ from HV in amygdala activation in this study. The second study used a parametric design and found that, during both implicit and explicit processing of face emotion, amygdala activation increased in HV as the degree of anger on a face increased, but such modulation did not occur in BD or SMD (Thomas et al., 2012). Of note, while BD and SMD did not differ in amygdala activation in this second study, the two groups differed in parametric modulation of posterior cingulate activation during processing of angry faces and in fronto-parietal activation during processing of happy faces. Taken together, this work finds complex differences among SMD, BD, and HV youth in amygdala function, with signs of both similarities and differences between SMD and BD youth.

Because of the inconsistent results of the two previous studies, the degree to which dysfunction in the amygdala or other regions is similar or different in SMD and BD during face-emotion processing warrants further research. The current study compared neural activity in SMD, BD, and HV youths using an implicit face emotion processing paradigm in which subjects are asked to identify the gender of fearful, angry, and neutral faces. We chose this paradigm because it has been used widely in BD research (Hassel et al., 2008; Kalmar et al., 2009; Lawrence et al., 2004; Shah et al., 2009; Surguladze et al., 2010) and appears to be particularly effective in eliciting differences in amygdala activation between patients with BD and healthy subjects (Chen et al., 2011; Lawrence et al., 2004; Phillips et al., 2008). Moreover, the current study addresses limitations in the preceding two studies. Specifically, the task in Brotman et al. (2010) was underpowered due to a limited number of replicates of each condition. In addition, neural responses to fearful faces have never been compared between BD, SMD, and HV although abnormal amygdala responses to fearful faces in BD have been found in several studies (Kalmar et al., 2009; Lawrence et al., 2004; Pavuluri et al., 2007).

We selected the amygdala as our region of interest (ROI) based on well-documented evidence of amygdala abnormalities in BD (Chen et al., 2011; Strakowski et al., 2012; Townsend and Altshuler, 2012). Indeed, using this paradigm and a largely overlapping sample, we compared youth with BD, adults with BD, and age-matched HV, and found that, compared to HV, BD youths exhibited amygdala hyperactivity across fearful, angry and neutral expressions. With regard to SMD, based on Brotman et al. (2010) and Thomas et al. (2012), we hypothesized that SMD would show abnormal amygdala responses to emotional and neutral expressions compared to HV. However, due to the differing results of the two prior SMD studies, we could not specify the directionality of the SMD vs. HV differences *a priori*, nor could we make a firm prediction as to whether SMD and BD would differ in amygdala activity.

In addition to an amygdala ROI analysis, we also conducted a whole-brain analysis. Thomas et al. (2012) found abnormal PCC activation in both SMD and BD during processing of angry faces. In addition, work in the same sample (Kim et al., 2012) and by other groups (Chang et al., 2004b; Pavuluri et al., 2007) suggest decreased activation in the PCC in BD vs. HV during processing of negative emotional stimuli. Based on these studies, we hypothesized that both SMD and BD would show decreased PCC activation vs. HV while processing angry and fearful expressions.

## 2 Materials and methods

### 2.1 Participants

The 53 participants included 19 BD youth, 19 SMD youth, and 15 HV youth. While none of the SMD data have been published previously, data have been published for 18 of the 19 BD youth and all HV youth (Kim et al., 2012). Participants enrolled in an Institutional Review Board approved protocol at the National Institute of Mental Health. Written informed consent and assent were acquired from parents and children, respectively. Patient participants were recruited through multiple sources including advertisements to local mental health professionals and advertisements on support group websites. HV youth were recruited through advertisements in the local area.

All participants were assessed for BD, SMD, and comorbidities using the Schedule for Affective Disorders and Schizophrenia for School-Age Children-Present and Lifetime version (K-SADS-PL) (Kaufman et al., 1997), including a module designed to ascertain SMD reliably (Leibenluft et al., 2003). The interview was administered separately to child and parent by a clinician, masters' level or above, with high inter-rater reliability (κ ≥ 0.9). Diagnoses, including comorbidities, were established based on consensus between the two clinicians in consultation with a psychiatrist.

BD children met the “narrow phenotype” criteria of BD (Leibenluft et al., 2003). These criteria required at least one full-duration (hypo)manic episode characterized by abnormally elevated mood, and three or more of the “B” mania symptoms in the DSM-IV-TR. The criteria for SMD required non-episodic, chronic irritability, defined as angry mood on most days, for at least half the day, noted by others, and developmentally inappropriate hyperreactivity to negative emotional stimuli manifest as emotional outbursts ≥ 3 ×/week. SMD criteria also required hyperarousal (characterized by ≥ 3 of the following symptoms: insomnia, distractibility, psychomotor agitation, racing thoughts/flight of ideas, pressured speech, and intrusiveness). All of these symptoms must have had onset before the age of 12, be present for ≥ 1 year with no symptom-free period longer than 2 months, and caused severe impairment in one of three settings (home, school, peers) and mild impairment in another (Leibenluft et al., 2003) (see Table 1).

HV youth were medication free, had no first-degree relatives with a mood disorder, and did not meet criteria for any diagnosis as assessed by the K-SADS-PL.

Mood state was assessed within 48 h of the scan for BD and SMD patients. Depressive symptomatology was assessed in both groups using the Children's Depression Rating Scale (CDRS) (Poznanski et al., 1979). Mania was assessed in BD youths using the Young Mania Rating Scale (YMRS) (Young et al., 1978). Children were not withdrawn from medication for the purpose of scanning because of the severity of their illness. However, data suggest that medication tends to be associated with Type II, rather than Type I, errors (Hafeman et al., 2012).

IQ was determined for all three groups using the Wechsler Abbreviated Scale of Intelligence (WASI) (Weschler, 1999). Participants were excluded if they had IQ < 70, history of head trauma, neurological disorder, pervasive developmental disorder, unstable medical illness, or substance abuse/dependence. A total of 84 scans were acquired, resulting in 53 usable scans. There were 31 scans excluded due to scanner/equipment malfunction (N = 17), excessive movement (N = 1), or low behavioral accuracy (< 65% correct responses; N = 13). The groups did not differ in percentage of excluded participants due to behavioral accuracy or movement (*p*s > .10).

## 3 fMRI Paradigm

This paradigm has been used previously (Blair et al., 2008; Kim et al., 2012; Marsh et al., 2008). Subjects viewed static grayscale images of emotional expressions of 10 men and women from the Pictures of Facial Affect series (Ekman and Friesen, 1976). Expressions were cropped to include the entire face. Stimuli included neutral, fearful, or angry expressions, with the last two types showing parametrically modulated intensity (50%, 100%, 150%) to enhance ecological validity and minimize blood-oxygen-level-dependent (BOLD) signal habituation. Since neutral expressions may appear threatening (Phillips et al., 2004), neutral and happy faces were morphed to create a 25% happiness expression, which is considered neutral by most subjects (Young et al., 1997). Participants indicated the gender (male, female) of each face using a two-button box. Each trial consisted of a single face presented for 2500 ms, followed by a 500 ms fixation cross, and each run consisted of 80 face trials. There were 25 randomly interspersed fixation trials (500 ms) per run in order to create jitter. Each of the four runs included 80 face trials (20 neutral trials and 10 trials of each intensity of fearful and angry faces) and 25 fixation trials. The trial order was randomized within each run.

## 4 Image acquisition

Data were acquired on a 1.5-T General Electric Signa scanner (Milwaukee, WI). Structural images used T1-weighted axial acquisition (three-dimensional spoiled-gradient-recall acquisition in the steady state with inversion recovery prep pulse; 128 1.5-mm axial slices, 256 × 256 matrix, 240 mm FOV, TR = 8.1 ms, TE = 3.2 ms, flip angle = 20^°^) were acquired to be coplanar with the functional scans for spatial registration. Functional imaging was performed axially using a multi-slice gradient echo-planar sequence (31 4-mm slices, voxels = 3.75 × 3.75 × 4 mm, 64 × 64 matrix, 240 mm FOV, TR = 3000 ms, TE = 30 ms, flip angle = 90^°^).

## 5 Data analysis

### 5.1 Behavior

We tested for group differences in accuracy and reaction time (RT) using two two-way repeated-measure analyses of variance (ANOVA) with group (BD, SMD, HV) as a between-subject factor and emotion (angry, fearful, neutral) as a within-subject factor in SPSS (SPSS, Inc., Chicago, Ill). Post-hoc t-tests were conducted using SPSS.

### 5.2 Imaging

#### 5.2.1 Data preprocessing

fMRI data were analyzed with the Analysis of Functional NeuroImages (AFNI) software package (Cox, 1996). The first four pre-steady-state volumes in each run were discarded. Preprocessing included slice timing correction, motion correction, image realignment, and the application of a 6 mm root-mean-square (RMS) deviation Gaussian blur. Images with motion greater than 2 mm in any direction were censored. If more than 5% of the total images were censored, a participant was excluded.

At the individual subject level, regressors for each emotion were created by convolving stimulus trains with a gamma-variate hemodynamic-response function. Fearful and angry regressors were weighted according to emotion intensity (1 for 50%, 2 for 100%, and 3 for 150%) as in prior work (Morris et al., 1996). Only correct trials were included. Linear regression modeling was performed per voxel with the following regressors: three emotion regressors, one regressor for all incorrect trials, a third-order baseline drift function, and motion parameter regressors. Fixation trials formed the baseline, and activation to each emotion was defined vs. fixation. Beta coefficients from the individual subject level were oriented to the standard space of Talairach and Tournoux, and then re-sampled to resolution of 3 mm^3^.

#### 5.2.2 Region of interest (ROI) analysis

Anatomic masks of the right and left amygdala were created based on the Talairach–Tournoux Daemon (Talairach and Tournoux, 1988). Mean percent signal change from each event type vs. fixation was averaged across all voxels in each amygdala mask, extracted using the AFNI program “3dROIstats,” and entered into SPSS. A two-way repeated-measure ANOVA with *Diagnosis* (BD, SMD, HV) as a between-subject factor and *Emotion* (angry, fearful, neutral) as a within-subject factor was performed in the right and left amygdala, using SPSS. Post-hoc t-tests were performed to identify group differences.

#### 5.2.3 Whole-brain analysis

A group-level two-way repeated-measure ANOVA with *Diagnosis* (BD, SMD, HV) as a between-subject factor and *Emotion* (angry, fearful, neutral) as a within-subject factor was conducted with GroupAna in AFNI. Using the 3dClustSim program in AFNI (http://afni.nimh.nih.gov/pub/dist/doc/program_help/3dClustSim.html), Monte Carlo simulation (10000 iterations, 54 × 64 × 50 dimensions, 3 × 3 × 3 voxels, 9 × 9 × 8 mm smoothness) indicated that an initial, voxel-wise threshold of *p* < 0.001 and a minimum cluster size of 24 voxels gave a corrected *p*-value of 0.05. In each suprathreshold cluster, mean percent signal change data from each event type vs. fixation was extracted using AFNI “3dROIstats” and entered in SPSS. Post-hoc analyses were performed in SPSS. Independent samples t-tests were performed to identify differences between groups and conditions. One-sample t-tests were performed to test whether the mean activation of each group differed significantly from zero.

#### 5.2.4 Post-hoc analyses on effects of mood state, medication, and comorbid illnesses

We conducted post-hoc exploratory t-tests in SPSS to test potentially confounding effects of mood state, medication, and comorbid illnesses on our results (see Appendix A). Because these exploratory post-hoc analyses included relatively small subsets of BD and SMD patients, we report significant results as well as those at a trend level, *p* < .10.

## 6 Results

### 6.1 Demographic and clinical characteristics

Groups did not differ on age, gender, or intelligence (Table 2). BD and SMD patients also did not differ on mood state, medication status, or comorbid illnesses except that SMD had a higher rate of conduct disorder (CD) or oppositional defiant disorder (ODD), *p* < .05.

### 6.2 Behavioral data

Table 3 provides descriptive statistics of behavior i.e., reaction time and accuracy. For reaction time, there was no *Diagnosis* × *Emotion* interaction, and no main effects. For accuracy, there was a main effect of *Emotion* (*F*(1,50) = 6.79, *p* < .05). The post-hoc analysis revealed that, across the three groups, accuracy for neutral expressions was higher than accuracy for fearful (*t*(52) = 2.65, *p* < .05) or angry (*t*(52) = 3.34, *p* < .005) expressions. There was no *Diagnosis* × *Emotion* interaction or main effect of Diagnosis.

### 6.3 fMRI data

#### 6.3.1 ROI analysis

**Fig. 1:**
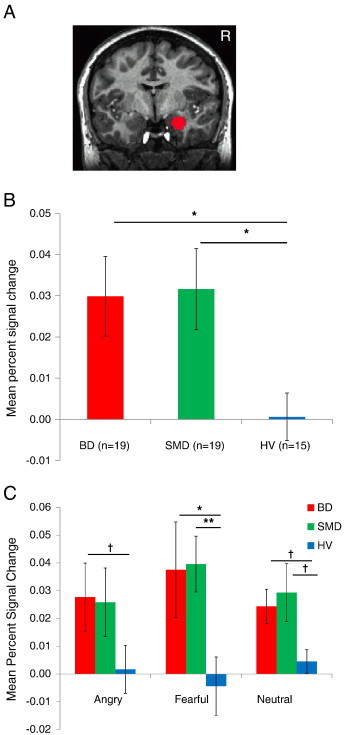
Findings of right amygdala ROI analysis. A. Right amygdala mask. B. Main effect of *Diagnosis* (*F*(2,50) = 3.39, *p* < .05). Mean percent signal changes across all emotion expressions in right amygdala. The error bars represent the Standard Error of the Mean percent signal changes. *p < .05. C. Activation of right amygdala for each expression vs. fixation. BD showed greater activation than HV in fearful expressions (*t*(32) = 2.52, *p* < .05) and in angry and neutral expressions at a trend level (*ts*(32) > 1.76, *ps* < .10). SMD showed greater activation than HV in fearful expressions (*t*(32) = 3.02, *p* < .01) and in neutral expressions at a trend level (*t*(32) = 2.00, *p* < .10). † p < .10, *p < .05, **p < .01.

In the right amygdala, a *Diagnosis* × *Emotion* interaction was not significant; however, there was a main effect of *Diagnosis* (*F*(2,50) = 3.39, *p* < .05). Post-hoc analyses of this main effect revealed that, similar to BD, SMD showed greater amygdala activation than HV across all expressions (*p* < .05; Fig. 1a,b). Activation did not differ between BD and SMD. There was no main effect of *Emotion*.

Although there was no *Diagnosis* × *Emotion* interaction, the activation of each expression vs. fixation was examined (Fig. 1c). BD showed greater activation than HV in fearful expressions (*t*(32) = 2.52, *p* < .05) and in angry and neutral expressions at a trend level (*ts*(32) > 1.76, *ps* < .10). SMD showed greater activation than HV in fearful expressions (*t*(32) = 3.02, *p* < .01) and in neutral expressions at a trend level (*t*(32) = 2.00, *p* < .10). There was no difference in BD and SMD. Thus, both BD and SMD showed relative hyperactivation vs. HV for all expressions.

In the left amygdala, no *Diagnosis* × *Emotion* interaction or main effect of *Diagnosis* was found. However, a main effect of *Emotion* was significant (*F*(2,100) = 3.92, *p* < .05), indicating that responses to fearful expressions were greater than responses to neutral expressions across groups (*p* < .01).

#### 6.3.2 Whole-brain analysis

There were *Diagnosis* × *Emotion* interactions in 7 areas: bilateral dorsal anterior cingulate cortex (ACC), bilateral posterior cingulate cortex (PCC), bilateral posterior insula, and left inferior parietal lobe (IPL) (Table 4). Post-hoc analyses revealed that these significant interactions were driven by differences between SMD and the other groups while viewing fearful expressions, and between BD and the other groups while viewing angry expressions.

**Fig. 2:**
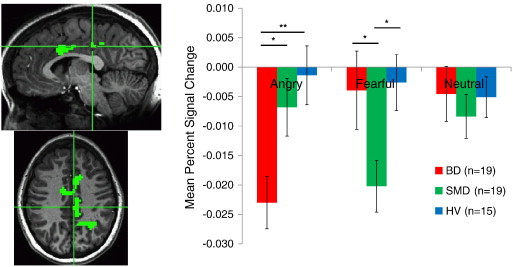
*Diagnosis* × *Emotion* interaction in left posterior cingulate cortex. In response to angry expressions, BD patients exhibited deactivation compared to SMD and HV youth. *p < .05, **p < .01. In response to fearful expressions, SMD patients exhibited deactivation compared to BD and HV youth.

**Fig. 3:**
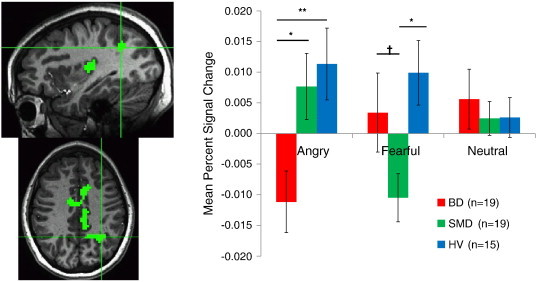
*Diagnosis* × *Emotion* interaction in left inferior parietal cortex/precunues. In response to fearful expressions, SMD patients exhibited deactivation compared to BD and HV youth. In response to angry expressions, BD patients exhibited deactivation compared to SMD and HV youth. † p < .10, *< .05, **< .01.

Specifically, while viewing fearful expressions, SMD, but not BD or HV, showed deactivation in the left PCC (Fig. 2), left IPL/precuneus (Fig. 3), and left posterior insula (*ps* < .10). In these regions, activations of BD and HV did not differ from zero.

In contrast, while viewing angry expressions, BD, but not SMD or HV, showed deactivation in the left dorsal ACC, left PCC (Fig. 2), left IPL/precuneus (Fig. 3), and bilateral posterior insula (*ps* < .05). In these regions, activations of SMD and HV to angry expressions did not differ from zero. In addition, in the right dorsal ACC, BD and HV showed significant deactivation when viewing angry faces, but activation in SMD did not differ from zero.

**Fig. 4:**
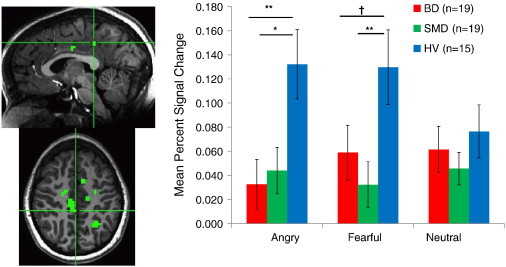
*Diagnosis* × *Emotion* interaction in right posterior cingulate cortex. In response to angry and fearful expressions, both BD and SMD patients exhibited decreased activation compared to HV youth.. † p < .10, *< .05, **< .01.

In the right PCC, while viewing angry and fearful expressions, both BD and SMD showed less activation than HV (*ps* < .10; Fig. 4). No between-group differences were identified in response to neutral expressions.

#### 6.3.3 Effects of mood state, medication, or comorbid illnesses

Post-hoc exploratory analyses revealed that the SMD vs. HV and BD vs. HV differences in the right amygdala remained significant at a trend level or better after controlling for comorbid illnesses, medication and mood state (*p*s < .08; Appendix A). Similarly, in most instances, group differences from the whole brain analysis remained significant at a trend level or better (*p*s < .10; Appendix A). The exception to this generalization is that, in the whole brain analysis, the group differences largely did not survive when BD with no comorbid ADHD (n = 9), or atypical antipsychotic-free BD youths (n = 6) were compared with other groups. Therefore, the effects of comorbid ADHD and atypical antipsychotic medication on the comparisons between BD and HV or SMD cannot be ruled out.

## 7 Discussion

We examined whether SMD and BD are associated with similar or different neural dysfunction during implicit processing of angry, fearful and neutral facial expressions. SMD and BD both demonstrated amygdala hyperactivity vs. HV across all three emotional expressions. However, there were *Diagnosis* × *Emotion* interactions in a number of other brain regions. Specifically, SMD differed from the other two groups in showing deactivation in response to fearful expressions in the PCC, IPL/precuneus and posterior insula. In contrast, BD differed from the other two groups in showing deactivation in response to angry expressions in these brain regions. BD also differed from SMD, but not HV, in showing ACC deactivation in response to angry faces. Thus, the current study, employing a well-powered task of implicit processing of angry, fearful and neutral expressions, revealed that BD and SMD exhibited similar amygdala abnormalities but unique neural dysfunction in regions mediating information integration and monitoring.

As hypothesized, SMD exhibited abnormal amygdala activity during implicit face emotion processing. Specifically, SMD showed amygdala hyperactivity across expressions, similar to BD. In BD and SMD, amygdala hyperactivation across emotions mirrors the face emotion labeling deficits observed in both disorders, since the labeling deficit is present across emotions (Guyer et al., 2007; Rich et al., 2008). Furthermore, generalized amygdala hyperactivity to a relatively wide range of emotional stimuli may be consistent with the fact that both SMD and BD are severely impairing mood disorders, with affected youth showing widespread deficits in their ability to respond appropriately to emotionally salient stimuli or contexts. Moreover, we previously reported that BD children showed greater amygdala hyperactivation across expressions than BD adults (Kim et al., 2012). Congruent with this, a recent meta-analysis suggested that face emotion labeling deficits are observed more consistently in youths than in adults with BD (Kohler et al., 2011). Thus, future research is needed to examine whether face emotion labeling deficits and amygdala hyperreactivity across a wide range of emotions are characteristic of pediatric but not adult BD, and whether these more widespread deficits are in turn associated with the more severe clinical course thought to occur in pediatric BD (Axelson et al., 2006; Birmaher et al., 2009).

Across studies comparing SMD and BD, findings suggest both similarities and differences between the groups, depending on the nature of cognitive processes engaged during face viewing. Two prior studies compared amygdala function in SMD and BD during face emotion processing. One of these, Thomas et al. (2012), also found a shared abnormality in amygdala activation between SMD and BD. The other, Brotman et al. (2010), used a task that differed significantly from the one here and found differences between SMD and BD in amygdala activity. However, both the current study and Brotman et al. (2010) found that SMD was associated with amygdala hyperactivity during implicit information processing of neutral expressions. The current study confirms and extends Brotman et al. (2010) by revealing amygdala hyperactivity in SMD during the implicit processing of angry and fearful, as well as neutral, expressions. The amygdala hyperreactivity to neutral expressions requires further research on whether both BD and SMD children recognize neutral expressions to be negative or hostile (Rich et al., 2006).

While we observed similar amygdala dysfunction in BD and SMD, the whole brain analysis revealed *Diagnosis* x *Emotion* interactions in a number of brain regions including the dorsal ACC, PCC, posterior insula, and IPL/precuneus. Specifically, BD differed from the other two groups in showing deactivation in response to angry faces in the PCC, posterior insula, and IPL/precuneus. On the other hand, SMD differed from the other two groups in showing deactivation in the same regions in response to fearful faces.

Prior research in a range of populations suggests that activity in these medial brain structures tends to vary in tandem, operating as part of a “default-mode” network (Raichle and Snyder, 2007). As such, the current findings suggest that emotion processing deficits in BD and SMD are associated with abnormal activity in some of the neural regions that comprise the default mode network. While disagreement persists concerning the precise functions of this network, data suggest that it is involved in the monitoring of internal states and in the shifting of attention toward or away from internal cues. In the current study, we found enhanced task-related deactivation in patients, suggesting aberrant modulation of interoceptive cues during tasks containing negative emotional stimuli. Indeed, we have previously reported activation differences between BD, SMD, and HV in these regions during other emotion information processing tasks. The one prior study in SMD that included a whole-brain analysis (Thomas et al., 2012) found abnormal modulation of PCC activity in SMD and BD in response to a face morphing from neutral to angry. In addition, a prior MEG study found decreased insula activation in BD, compared to SMD and HV, in the context of a frustrating (i.e., potentially anger-inducing) paradigm (Rich et al., 2011).

Our finding of possible aberrations in default mode network modulation should be viewed in the context of prior reports in childhood psychopathology that also reported between-group differences in levels of deactivation below an implicit baseline (Peterson et al., 2009). However, while we found *enhanced* deactivation in pediatric emotional disorders, Peterson et al. (2009) found *reduced* deactivation in pediatric behavioral disorders, albeit during a different cognitive task. This prior finding, combined with the emotion-specific differences we saw between BD and SMD in the current study, suggests the potential for disorder-specificity in default-mode perturbations. In addition, whereas we found between-group differences in the precuneus, posterior insula, and left PCC, Peterson et al. (2009) found prominent between-group differences in the ventral ACC. Thus, these findings suggest that regional-anatomical specificity in default-mode perturbations also may exist across disorders.

At the whole brain level, we found prominent abnormalities in BD in response to angry faces and in SMD in response to fearful faces. Such emotion-specific neural dysfunction is a novel finding that requires replication. Indeed, this emotion specificity is somewhat surprising because the behavioral studies documenting emotion identification deficits in SMD and BD do not find emotion specificity (e.g., (Guyer et al., 2007; Rich et al., 2008)). Focusing on BD only, where considerably more work has been done than in SMD, both a recent meta-analysis (Chen et al., 2011) and a recent review (Townsend and Altshuler, 2012) do not provide strong support for emotion-specific dysfunction in the amygdala or other brain regions, although both task design and patient characteristics differ among studies. In addition, it is challenging to design an fMRI task that has adequate trial numbers of a variety of emotions and is brief enough for patients, particularly pediatric patients, to tolerate. Therefore, the question of whether neural deficits in BD and/or SMD are emotion-specific has not been well-studied and is an important area for future research.

The comorbidity of ADHD in our BD and SMD subjects is extremely high i.e., 63% of BD and 79% of SMD also had an ADHD diagnosis. Regarding BD, the rate of ADHD in our sample is consistent with other imaging studies in the literature (Adleman et al., 2011; Brotman et al., 2010; Chang et al., 2004a; Deveney et al., 2012; Kim et al., 2012; Passarotti et al., 2010a, b; Thomas et al., 2012). Regarding SMD, the rate of ADHD is high by definition. That is, as noted in the Introduction and Table 1, SMD was operationalized to facilitate research on children with severe, chronic irritability and hyperarousal symptoms, since there is controversy in the literature as to whether such children should be considered to have BD. Since SMD youth were being given the diagnosis of BD in part because of overlap between the symptoms of ADHD and the “B” symptoms of mania, there will necessarily be high rates of ADHD in our SMD youth. While exploratory post-hoc analyses in the BD sample suggest that our amygdala finding is not due to the impact of comorbid ADHD, overall we cannot rule out the impact of ADHD on our findings. To do so, we would need a larger group of BD without ADHD as well as a sample of non-irritable youth with ADHD (Brotman et al., 2010); this is clearly an important focus for future research.

## 8 Conclusions

Our findings suggest that implicit face emotion processing in BD and SMD may elicit similar amygdala dysfunction, but unique neural dysfunction in information integration and monitoring regions. Specifically, SMD showed amygdala hyperactivity across angry, fearful and neutral expressions, similar to what has been documented previously in BD (Kim et al., 2012). However, in the PCC, IPL and posterior insula, only SMD showed deactivation in response to fearful expressions, while only BD showed deactivation in response to angry expressions. Amygdala hyperactivity in SMD and BD may be one underlying neural mechanism contributing to behavioral deficits in emotion recognition in both SMD and BD. However, group differences in activation in the posterior cingulate, posterior insula, and IPL suggest that additional neuroimaging studies are needed to specify the nature of the dysfunction in these regions across emotions and across patient groups, and to continue to test the extent to which the symptoms of SMD and BD are mediated by similar or different pathophysiologic mechanisms. Given that SMD and BD have different clinical developmental trajectories, it will be important to conduct longitudinal fMRI studies, guided by cross-sectional ones such as this, to elucidate the development of emotion dysregulation in SMD vs. BD and allow the integration of clinical and brain-based developmental measures.

## Figures and Tables

**Table 1:** Diagnostic criteria for severe mood dysregulation.

Inclusion criteria	Exclusion criteria
1. Aged 7–17, with the onset of symptoms before age 12. 2. Abnormal mood (specifically anger or sadness), present at least half of the day most days, and of sufficient severity to be noticeable by people in the child's environment (e.g., parents, teachers, peers). 3. Hyperarousal, as defined by at least three of the following symptoms: insomnia, agitation, distractibility, racing thoughts or flight of ideas, pressured speech, intrusiveness. 4. Compared to his/her peers, the child exhibits markedly increased reactivity to negative emotional stimuli that is manifest verbally or behaviorally. For example, the child responds to frustration with extended temper tantrums (inappropriate for age and/or precipitating event), verbal rages, and/or aggression toward people or property. Such events occur, on average, at least three times a week. 5. The symptoms noted in 2–4 above are currently present and have been present for at least 12 months without any symptom-free periods exceeding two months. 6. The symptoms are severe in at least one setting (i.e., violent outbursts, assaultiveness at home, school, or with peers). In addition, there are at least mild symptoms (distractibility, intrusiveness) in a second setting.	1. The individual exhibits any of these cardinal bipolar symptoms: elevated or expansive mood, grandiosity or inflated self-esteem, episodically decreased need for sleep. 2. The symptoms occur in distinct periods lasting more than 1 day. 3. Meets criteria for schizophrenia, schizophreniform disorder, schizoaffective illness, pervasive development disorder, or PTSD. 4. Meets criteria for substance use disorder in the past 3 months. 5. IQ < 70 6. The symptoms are due to the direct physiological effects of a drug of abuse, or to a general medical or neurological condition.

Adapted from Rich et al. (2007).

**Table 2:** Demographic and clinical characteristics of bipolar disorder (BD), severe mood dysregulation (SMD) or healthy volunteer (HV) participants.

Characteristic	BD (N=19)	SMD (N = 19)	HV (N = 15)
	Mean (SD)	Mean (SD)	Mean (SD)
Age (years)	14.22 (2.53)	13.42 (2.63)	14.98 (2.03)
Wechsler Abbreviated Scale of Intelligence full-scale IQ^a^	104.58 (13.36)	103.89 (14.39)	108.27 (15.35)
Young Mania Rating Scale score (YMRS)^b^	6.28 (3.91)	–	–
Children's Depression Rating Scale Score (CDRS)^c^	26.53 (7.41)	27.06 (4.62)	–
Number of medications	3.00 (1.86)	2.05 (1.65)	–
Number of co-existing diagnoses	2.37 (1.16)	2.16 (1.38)	–

	N (%)	N (%)	N (%)

Male	10 (52.6)	10 (52.6)	5 (33.3)
BD I	14 (73.7)	–	–
BD II	5 (26.3)	–	–
Mood state^d^			
Euthymic	16 (84.2)	19 (100)	–
Depressed	2 (10.5)	0 (0)	–
Manic or hypomanic	1 (5.3)	–	–
Mixed	0 (0)	–	–
co- existing diagnoses			
ADHD	12 (63.2)	15 (78.9)	–
Oppositional defiant disorder or conduct disorder^e^	3 (15.8)	9 (47.4)	–
Anxiety disorder	10 (52.6)	6 (31.6)	–
Medication			
Unmedicated	3 (15.8)	4 (21.1)	–
Atypical antipsychotic	13 (68.4)	8 (42.1)	–
Lithium	5 (26.3)	2 (10.5)	–
Antiepileptic	12 (63.2)	7 (36.8)	–
Antidepressant	9 (47.4)	4 (21.2)	–
Stimulant	6 (31.6)	8 (42.1)	–

BD = bipolar disorder, SMD = severe mood dysregulation, HV = healthy volunteer.

a For two-scale IQ N = 4; for full-scale IQ N = 53.

b Data missing for one BD patient.

c Data missing for one BD and one SMD patient. The Structured Interview Guide for the Hamilton Depression Rating Scale, Seasonal Affective Disorders (SIGH-SAD) was collected instead of CDRS for one BD patient (score = 13) and one SMD patient (score = 13) who were over 18 at the time of the scan.

d For one BD patient and one SMD patient who were over 18, SIGH-SAD was used instead of CDRS to determine mood state. Euthymia was defined as: CDRS < 40 (or SIGH-SAD ≤ 20) and YMRS ≤ 12. Depression was defined as: CDRS ≥ 40 (or SIGH-SAD > 20) and YMRS ≤ 12. Mania/hypomania was defined as: CDRS < 40 (or SIGH-SAD ≤ 20) and YMRS > 12. Mixed state was defined as: CDRS ≥ 40 (or SIGH-SAD > 20) and YMRS > 12.

e BD vs SMD [χ ^2^ (1, N = 38) = 4.39, p < .05].

**Table 3:** Behavioral performance of youths with bipolar disorder (BD), severe mood dysregulation (SMD) or healthy volunteer (HV) participants.

Condition	BD(N = 19)	SMD (N = 19)	HV (N = 15)
	Mean ± SD	Mean ± SD	Mean ± SD
Percent correct			
Angry expressions	85.65 ± 9.87	83.88 ± 9.75	90.92 ± 8.82
Fear expressions	85.94 ± 8.80	84.59 ± 10.11	91.49 ± 9.51
Neutral expressions	87.41 ± 11.15	86.09 ± 9.28	93.25 ± 7.45
Reaction time			
Angry expressions	937.95 ± 108.76	904.07 ± 159.62	843.52 ± 152.43
Fear expressions	936.50 ± 111.50	902.17 ± 147.25	830.77 ± 161.30
Neutral expressions	940.10 ± 118.14	893.00 ± 162.98	827.35 ± 154.40

BD = bipolar disorder, SMD = severe mood dysregulation, HV = healthy volunteer.

**Table 4:** Areas showing *Diagnosis* × *Emotion* interactions with post-hoc analyses of between-group differences in angry, fearful, and neutral expressions.

Area of Activation	Brodmann area	Side	Cluster size	Talairach coordinates			*F* (2, 50)	Between-group differences
				x	y	z		
Anterior cingulate gyrus	24	L	149	− 4	11	32	8.42	*Angry*: BD<SMD⁎⁎, BD<HV⁎
Anterior cingulate gyrus	24	R	27	5	11	32	5.39	*Angry*: BD<SMD⁎⁎
Posterior cingulate gyrus	31	L	65	− 4	− 28	35	5.68	*Angry*: BD<SMD⁎, BD<HV⁎⁎ *Fearful*: SMD<BD⁎, SMD<HV⁎
Posterior cingulate gyrus	31	R	30	2	− 34	41	5.17	*Angry*: BD<HV⁎⁎, SMD<HV⁎ *Fearful*: BD<HV† SMD<HV⁎⁎
Posterior insula	13	L	45	− 37	− 22	14	5.19	*Angry*: BD<SMD⁎, BD<HV⁎ *Fearful*: SMD<BD⁎, SMD<HV⁎
Posterior insula	13	R	92	35	− 16	11	5.28	*Angry*: BD<SMD⁎⁎, BD<HV⁎⁎
Inferior parietal lobe	40	L	69	− 31	− 52	35	6.32	*Angry*: BD<SMD⁎, BD<HV⁎⁎ *Fearful*: SMD<BD†, SMD<HV⁎⁎

p < .05 (corrected). BD = bipolar disorder, SMD = severe mood dysregulation, HV = healthy volunteer. x,y, and z coordinates refer to the voxel with maximum signal intensity. Note: The post-hoc analyses of angry, fearful, neutral expressions were conducted in regions identified by the primary *Group* × *Emotion* ANOVA analysis.

† p < .10.

⁎ p < .05.

⁎⁎ p < .01.
