# Supplementary material for: Elevated amygdala responses to emotional faces in youths with chronic irritability or bipolar disorder
Source: Neuroimage Clin. 2013 Apr 21;2:637–45. doi: 10.1016/j.nicl.2013.04.007 (PMC3746996; doi:10.1016/j.nicl.2013.04.007)
Supplement: Supplementary material [file mmc1.docx]

Appendix A.

**Effects of mood state, medication, and comorbid illnesses**

Post-hoc analyses examined whether clinical differences between BD, SMD and HV youths might account for the main effect of diagnosis in the right amygdala and the group x emotion interaction identified in 7 brain regions.

A series of post-hoc t-tests in SPSS were performed to compare neural activity in regions identified by the primary ANOVA analyses (Table s1). In BD youths, first, to examine the effects of comorbid illnesses, we compared (1) BD without comorbid anxiety disorders (N=9) vs. HV; (2) BD without ODD or CD (N=16) vs. HV; (3) BD without ADHD (N=7) vs. HV youths. Second, to test the effects of mood state, euthymic BD (N=15) were compared with HV. Third, since the number of unmedicated BD youths was very small (N=3), we could not compare them to HV youths. However, we examined the effect of each medication class in BD by comparing (1) antidepressant-free BD (N=10) vs. HV; (2) antiepileptic-free BD (N=7) vs. HV; (3) stimulant-free BD (N=13) vs. HV youths; (4) lithium-free BD (N=14) vs. HV youths; and (5) atypical antipsychotic-free BD (N=6) vs. HV youths.

Similar exploratory post-hoc analyses compared SMD and HV youths (Table s1). First, to examine the effects of comorbid illnesses, we compared (1) SMD without comorbid anxiety disorders (N=13) vs. HV; (2) SMD without ODD or CD (N=10) vs. HV. Given the small number of SMD without ADHD (N=4), we were unable to test the potential impact of ADHD in the SMD findings. Second, since the number of unmedicated SMD youths was also very small (N=4), we examined the effect of each medication class in SMD by comparing (1) antidepressant-free SMD (N=15) vs. HV; (2) antiepileptic-free SMD (N=12) vs. HV; (3) stimulant-free SMD (N=11) vs. HV youths; (4) lithium-free SMD (N=17) vs. HV youths; and (5) atypical antipsychotic-free SMD (N=11) vs. HV youths.

*ROI analysis*

In all comparisons, BD youths showed right amygdala hyperactivity vs. HV youths (*ps* < .05). Similarly, in all comparisons, SMD showed at least a trend toward right amygdala hyperactivity vs. HV youths (*ps* < .10). Thus, right amygdala hyperactivity in both BD and SMD youths did not appear to be driven by mood state, medication, or comorbid illnesses.

*Whole-brain analysis*

The between-group differences from the whole-brain analysis survived most comparisons at at least the trend level (all p’s < .10) with two exceptions. Specifically, the differences did not remain significant when BD youths with no comorbid ADHD (n=9), or atypical antipsychotic-free BD youths (n=6) were compared with other groups; of note, these comparisons are underpowered. Nonetheless, the effects of comorbid ADHD and atypical antipsychotic medication on BD vs. HV or SMD differences in the regions from the whole-brain analysis cannot be ruled out.

Table s1. Post-hoc analysis of effects of comorbidity, mood state, medication on findings from the ROI analysis and the whole-brain analysis.

| **Between-group differences** | **Area of Activation** | **Condition** | **Comorbid illnesses** | | | **Mood State** | **Medication** | | | | |
| --- | --- | --- | --- | --- | --- | --- | --- | --- | --- | --- | --- |
|  |  |  | no comrbid anxiety | no comorbid ODD or CD | no comorbid ADHD | euthymic | antidepressant-free | antiepileptic-free | stimulant-free | lithium-free | atypical antipsychotic-free |
|  |  |  | BD (n=9) | BD (n=16) | BD (n=9) | BD (n=15) | BD (n=10) | BD (n=7) | BD (n=13) | BD (n=14) | BD (n=6) |
|  |  |  | SMD (n=13) | SMD (n=10) | SMD (n=4)^a^ | SMD (n=19)^b^ | SMD (n=15) | SMD (n=12) | SMD (n=11) | SMD (n=17) | SMD (n=11) |
| **ROI analysis** |  |  |  |  |  |  |  |  |  |  |  |
| BD > HV | Right Amygdala | *across all expressions* | * | * | ** | * | * | * | * | * | * |
| SMD > HV | Right Amygdala | *across all expressions* | † | † | -- | -- | * | **** | * | *** | * |
|  |  |  |  |  |  |  |  |  |  |  |  |
| **Whole-brain analysis** | |  |  |  |  |  |  |  |  |  |  |
| BD < HV | Left ACC | *Angry* | * | * | n.s. | † | n.s. | n.s. | † | n.s. | n.s. |
|  | Left PCC | *Angry* | *** | *** | † | ** | * | ** | * | * | † |
|  | Left PCC | *Fearful* | * | * | -- | -- | ** | * | † | * | * |
|  | Right PCC | *Angry* | ** | * | * | ** | * | * | n.s. | * | * |
|  | Left Insula | *Angry* | * | ** | n.s. | ** | † | † | † | n.s. | n.s. |
|  | Right Insula | *Angry* | * | ** | † | *** | * | * | * | * | n.s. |
|  | Left IPL | *Angry* | *** | ** | n.s. | * | * | *** | * | * | n.s. |
|  |  |  |  |  |  |  |  |  |  |  |  |
| SMD < HV | Right PCC | *Angry* | *** | ** | -- | -- | * | * | *** | * | * |
|  | Right PCC | *Fearful* | * | * | -- | -- | * | ** | † | * | † |
|  | Left Insula | *Fearful* | * | * | -- | -- | * | † | n.s. | * | * |
|  | Left IPL | *Fearful* | *** | * | -- | **** | * | * | * | ** | ** |
|  |  |  |  |  |  |  |  |  |  |  |  |
| BD < SMD | Left ACC | *Angry* | * | ** | -- | * | † | * | * | * | n.s. |
|  | Right ACC | *Angry* | ** | * | -- | *** | * | * | *** | * | n.s. |
|  | Left PCC | *Angry* | † | n.s. | -- | * | n.s. | † | † | † | n.s. |
|  | Left Insula | *Angry* | † | * | -- | *** | * | * | * | † | n.s. |
|  | Right Insula | *Angry* | ** | *** | -- | ** | * | *** | ** | ** | n.s. |
|  | Left IPL | *Angry* | * | † | -- | * | † | ** | † | † | n.s. |
|  |  |  |  |  |  |  |  |  |  |  |  |
| SMD < BD | Left Insula | *Fearful* | † | † | -- | * | * | * | n.s. | * | * |

^†^p< .10, *p<.05, **p<.01, ***p<.001

BD = bipolar disorder, SMD = severe mood dysregulation, HV = healthy volunteer, n.s. = not significant (*p* > .10)

^a^ because SMD without ADHD comorbid was too few (n=4), post-hoc analysis was not performed.

^b^because all youths in the SMD group were euthymic, post-hoc analysis was not performed.
